# Do African and European ancestry and polymorphism of HLA class I play an important role in controlling HTLV-1 proviral load in admixed cohorts from Salvador, Brazil?

**DOI:** 10.1186/1742-4690-11-S1-P76

**Published:** 2014-01-07

**Authors:** Viviana Nilla Olavarria, Eduardo José Melo dos Santos, Sidney Emanuel Batista dos Santos, Fernanda Grassi, Ramon Kruchewesky, Luís Cristóvão de Moraes Sobrino Pôrto, Xiaojan Gao, Mary Carrington, Bernardo Galvão-Castro

**Affiliations:** 1 HTLV Reference Center, Bahiana School of Medicine and Public Health, Brazil; 2 Human and Medical Genetics, Federal University of Pará, Brazil; 3 Advanced public Health Laboratory, Fiocruz, Ba, Brazil; 4 Laboratory of Histocompatibility and Cryopreservation, State University of Rio de Janeiro, Brazil; 5 Laboratory of Genomic Diversity, National Cancer Institute, Frederick, Maryland, USA

## 

HAM/TSP is one of the most prevalent clinical manifestations of HTLV- 1 infection, likely related to high proviral load (PVL). We investigated HLA-A, -B and –C polymorphisms and determined the individual ancestry proportion of European, African and Amerindian in 209 HTLV-1 infected individuals in order to identify genetic factors that associate with HAM/TSP. The ancestry estimation using the STRUCTURE program considered the genotyping of 47 SNP ancestry markers. Using rigorous clinical and laboratory criteria, we defined two subsets: HAM/TSP (41 patients) and asymptomatic (168). No associations were found between HLA polymorphisms and HAM/TSP. The European ancestry in the HAM/TSP sample (43%) was 7% lower than in the asymptomatic group (50%), while the African ancestry was 6% higher among HAM/TSP patients (45%) than among asymptomatics (39%). The Amerindian ancestry was quite similar for both HAM/TSP (12%) and asymptomatic (11%) samples. Although these differences did not reached statistical significance, they provide interesting clues to be more deeply explored. There was no correlation between PVL and individual European, African and Amerindian ancestry estimates when considering the entire sample. However, considering only the HAM/TSP subsample, our results suggest that European ancestry may predispose to higher PVL (Spearman´s correlation; rs= 0.3611; p=0,0422), while African ancestry associates with lower PVL (rs= -0.3875; p=0,0283). These results suggest that once HAM/TSP develops, people of African ancestry may control PVL to a greater extent. More efficient PVL control among people of African descent may be the result of selection pressure occurring over the long history of co-evolution of HTLV-1 and its human host in Africa.

